# Nutritional nursing competence of clinical nurses and its influencing factors: a cross-sectional study

**DOI:** 10.3389/fnut.2024.1449271

**Published:** 2024-12-18

**Authors:** Yuan Tang, Xiumei Wen, Xiaoli Tang, Xiaoxue Li, Li Zhang, Shujuan Duan, Ping Long, Zixuan Zhou

**Affiliations:** ^1^Neurosurgery Center Department, Sichuan Clinical Research Center for Cancer, Sichuan Cancer Hospital & Institute, Sichuan Cancer Center, Affiliated Cancer Hospital of University of Electronic Science and Technology of China, Chengdu, China; ^2^Yanjiang District People’s Hospital, Ziyang, China

**Keywords:** nutritional nursing, clinical nurses, competence, influencing factors, cross-sectional study

## Abstract

**Objective:**

Assessing the nutritional nursing competence of clinical nurses will enable nursing managers to develop measures to effectively address the nutritional nursing needs of patients. Therefore, the purpose of this study was to investigate the status quo and influencing factors of nutritional nursing competence of nurses in China.

**Method:**

A cross-sectional survey was conducted among more than 1300 clinical nurses from 10 provinces in eastern, central and western China by using self-designed socio-demographic questionnaire and nutrition nursing ability scale compiled by Zhu Xinyi.

**Result:**

The median score of nurses’ nutritional nursing ability was 238 (210, 258). Univariate analysis confirmed that a total of 12 factors were shown to be statistically significant for nurses’ competency in nutritional care. The results of multiple linear regression analysis showed that department, hospital level, monthly income, learned nutrition courses, regular participation in nutrition continuing education and training, be a nutrition specialist nurse, participation in nutrition nursing knowledge and skills training, and the hospital’s regular conduct of special quality supervision of nutrition nursing and carried out special quality improvement projects of nutrition nursing.

**Conclusion:**

In China, the nutritional nursing competence of nurses is at an upper medium level and needs to be further improved. In order to improve the level of nutrition nursing, encourage and support nurses to take targeted nutrition care education and training, nursing managers should also adopt multi-mode intensive inspection and examination.

## 1 Introduction

Good nutrition plays a vital role in a patient’s recovery. Studies have shown that the proportion of patients suffering from malnutrition in hospitals is 8.8% ~ 60% (1–3). The reason was that most patients received inadequate nutrition sources and supplement channels during the hospital period, and nearly half of the patients received superficial nutrition care guidance at discharge (4). The effects of malnutrition on a patient’s body are devastating, such as increased risk of infection (5–8), poor wound healing (9, 10), immune dysfunction (5, 11–13), sepsis (14–16)and more. Malnutrition causes unnecessary suffering, reduced recovery rates, prolonged hospital stays, and even death, as well as a significant burden on medical data (17–19).

How to reduce the incidence of malnutrition and ensure the reasonable nutrition of patients, nurses play a particularly important role in it. Nutritional support therapy requires a multidisciplinary nutritional therapy team involving doctors, dietitians, pharmacists, rehabilitators and nurses, among which clinical nurses are the executive body of the multidisciplinary nutritional therapy team (20, 21). It includes dietary behavior survey, nutritional risk screening, malnutrition assessment, nutrition health education, diet guidance, implementation of nutritional treatment, prevention and treatment of adverse reactions to nutritional treatment, weight monitoring, and promotion of scientific knowledge of nutrition (22–24). Accurate and standardized nutrition nursing is an important guarantee to ensure the safety and effectiveness of nutrition treatment. Nurses make a comprehensive assessment of patients’ nutritional status, work with physicians, dietitians and rehabilitators to develop personalized nutrition plans, and are responsible for implementing these plans to ensure that patients receive appropriate nutritional support (24, 25). At the same time, nurses are responsible for educating patients and their families about healthy eating, monitoring changes in patients’ nutritional status, and adjusting nutrition plans as needed (26–28). Their expertise and practice are essential to facilitate patient recovery and health maintenance. Nurses are important executors of health education in hospitals and communities, and bear great responsibility for nutrition and health education of patients and their families (29). Therefore, when faced with potential malnutrition or significantly impaired nutritional status, professional nursing staff are needed to undertake the work of nutrition knowledge promotion and nutrition health education. At present, many nurses in specialized nursing fields have been developed in China, but the specialty of nutrition nursing started late and is still in the stage of exploration and development. The training system of nutrition nursing specialist nurses has not been established in China, there are few nutrition specialist nurses, and the nutrition nursing competence of nurses is uneven.

Nutritional nursing competence refers to the comprehensive ability of nurses to carry out accurate nutritional risk screening, dietary assessment and nutritional evaluation, and correctly implement dietary guidance and nutritional support for patients in order to effectively improve their nutritional status and promote their health in clinical nursing work. As a result of growing awareness of the importance of nutritional care in the development of a patient’s disease, tools have been developed to assess the nutritional care competence of nurses. At present, it is not clear about the status quo of nutritional nursing competence of Chinese nurses, especially the sensitive factors that affect nurses’ nutritional care in busy clinical work are poorly understood. Therefore, the purposes of our exploatory, descriptive study were (1) to describe the current status of clinical nurses’ nutritional nursing competence and (2) to explore the influencing factors of nutritional nursing competence.

## 2 Methods

### 2.1 Design and setting

A cross-sectional study based on previous research was used, involving the completion of a self-reported questionnaire of approximately 20 min. Data was collected from November 2022 to March 2023 at 162 hospitals across 10 provinces in China.

### 2.2 Participants

The participants were 1,300 nurses engaged in clinical care from different hospitals in different regions, administrative and logistics nurses were not included. Convenient sampling was conducted in 10 provinces in East, Central, and Western China, including Shanghai Dongfang Hospital, Jiaxing Second People’s Hospital, Chaotian Dafeng Hospital, Jiangmen Maternal and Child Health Hospital, Luohe Central Hospital, Wuhan Integrative Orthopedic Hospital, JinChang People’s Hospital, HanZhong Railway Central Hospital, Sichuan Provincial Cancer Hospital, Ziyang People’s Hospital, Panzhihua Central Hospital and Chifeng People’s Hospital。According to Kendall’s sample size calculation principle, the sample size is 5 ~ 10 of the variable (30). There were a total of 78 variables, 15 related to general sociodemographic information,and 63 in the Nutrition Care Competence Scale. Consider 20% dropout, the total sample size is 488–975[78 × 5 × (1 + 0.2) = 488 78 × 10 × (1 + 0.2) = 975]. The criteria for inclusion were: (1) working in the hospital for at least 1 year, (2) being a registered nurse, and (3) agreeing to participate and signing an informed consent form. The exclusion criteria are: (1) nurses who are pregnant, nursing or on leave, and (2) nurses who are in administrative or logistical support positions. These instruments included two questionnaires, as follows.

#### 2.2.1 Self-designed socio-demographic characteristics questionnaire

There are 15 variables in this part, including gender, age, education background, professional title, working years, hospital level, department, monthly income, region, whether to learn nutrition courses in school, whether to participate in nutrition related continuing education and training, whether to be a nutrition specialist nurse, when to participate in nutrition nursing related knowledge/skills competition, and whether the hospital regularly carries out special examination of nutrition nursing.

#### 2.2.2 Nutritional care competence scale

The nutrition nursing competence scale of clinical nurses was compiled by Zhu Xinyi (31). On the basis of literature analysis and semi-structured interview, the evaluation index of nutrition nursing ability of clinical nurses was constructed through 2 rounds of letter consultation with 16 experts in Delphi, and the predictive test scale was compiled according to this. The scale was used to investigate the status quo of nutritional nursing competence of clinical nurses, and the confirmatory factor analysis and reliability and validity analysis were carried out to form a formal scale. The scale, a total of 63 entries, including professional knowledge, nutrition assessment of clinical nutrition, nutrition health education ability, nutritional support nursing ability and nutrition related quality five dimensions. It is scored on a 5-point Likert-type, with 1 to 5 points from “completely inconsistent” to “completely consistent.” The higher the equal score, the better the nutritional nursing competence. The overall Cronbach’s *α* was 0.991, the split half reliability was 0.926, and the test–retest reliability was 0.876 (*p* < 0.001) (22).

### 2.3 Data collection

A team of five registered nurses was assembled and trained in the details of the study. The training included the use of uniform instructions and the issuance of an electronic questionnaire to the subjects via the wechat platform. After the purpose, benefits, and risks of the study were clearly explained to each hospital and to each participant, we conducted this cross-sectional study with the consent of the participants. Participants answered anonymously and voluntarily, and could only submit the questionnaire after completing it. All participants are guaranteed confidentiality and informed that they can withdraw at any time. This questionnaire can only be filled out once with the same IP address, computer or mobile phone.

### 2.4 Data analysis

The social demographic data and nutritional nursing competence scores of clinical nurses were analyzed by descriptive statistics. The counting data were represented by frequency and percentage, while the measurement data did not conform to normality and homogeneity of variance, so *M* (*P_25_*, *P_75_*) was used to describe the data. Factors affecting nursing nutrition competence were analyzed by Mann–Whitney U test or Kruskall-Wallis H test. Multiple linear regression was used to identify important predictors between sociodemographic factors and clinical nurses’ nutritional nursing competence. All variables are entered step by step. The statistical significance was set at *p* < 0.05 for all tests.

## 3 Result

### 3.1 Participant characteristics

A total of 1,300 clinical nurses were investigated in this study. After double verification and screening, 1,218 valid questionnaires were obtained, and the effective rate of the questionnaire was 93.7%. Among 1,218 subjects, 95% were female and 85.6% were under 40 years old. Most of them had bachelor’s degree (78.7%), and 40.6% of them were supervisor nurses. Additional details are shown in Table 1.

**Table 1:** Comparison of various socio-demographic and job characteristics of clinical nurses’ nutritional nursing competence (*N* = 1,218).

Characteristic	Categories	Total sample, *N* (%)	*Z*	*p*
Gender	Male	61 (5)	−0.99	0.322
	Female	1,157 (95)		
Age	<30	469 (38.5)	2.029	0.566
	30 ~ 39	574 (47.1)		
	40 ~ 49	150 (12.3)		
	≥50	25 (2.1)		
Education background	College or below	251 (20.6)	18.728	<0.001
	Undergraduate	958 (78.7)		
	Master or above	9 (0.7)		
Professional title	Nurse	196 (16.1)	9.676	0.022
	Junior nurse	495 (40.6)		
	Registered nurse	455 (37.4)		
	Senior nurse	72 (5.9)		
Working years	<10 years	628 (51.6)	1.696	0.638
	10 ~ 19 years	453 (37.2)		
	20 ~ 29 years	111 (9.1)		
	≥30 years	26 (2.1)		
Hospital level	Second class B and below	76 (6.2)	82.935	<0.001
	Second class A	169 (13.9)		
	Third class B	222 (18.2)		
	Third class A	751 (61.7)		
Department	Internal medicine	365 (30)	31.788	<0.001
	Surgical department	315 (25.9)		
	Gynecology and obstetrics	64 (5.3)		
	Pediatrics department	63 (5.2)		
	ICU	188 (15.4)		
	Other	223 (18.3)		
Monthly income	≤3,000	155 (12.7)	26.406	<0.001
	3,001 ~ 5,000	470 (38.6)		
	5,001 ~ 8,000	444 (36.5)		
	>8,000	149 (12.2)		
Region	East region	262 (21.5)	40.109	<0.001
	Central region	444 (36.5)		
	West region	510 (41.9)		
Learn nutrition courses in school	Yes	633 (52)	−7.311	<0.001
	No	585 (48)		
Participate in nutrition related continuing education and training	Yes	487 (40)	−12.766	<0.001
	No	731 (60)		
Be a nutrition specialist nurse	Yes	47 (3.9)	−5.77	<0.001
	No	1,171 (96.1)		
Participate in nutrition nursing related knowledge/skills competition	Yes	137 (11.2)	−6.809	<0.001
	No	1,081 (88.8)		
Hospital regularly carries out special examination of nutrition nursing	Yes	540 (44.3)	−12.821	<0.001
	No	678 (55.7)		
Carry out nutrition and nursing special quality improvement projects	Yes	618 (50.7)	−12.994	<0.001
	No	600 (49.3)		

### 3.2 Nutritional nursing competence score of nurses

Table 2 shows the total score and the scores of each dimension of nutritional nursing competence of clinical nurses. The total score of nutrition nursing competence was 238 (210, 253), of which the score of nutrition professional knowledge was 25 (22, 28), clinical nutrition assessment competence was 37 (27, 40), nutrition health education competence was 48 (40, 52), nutrition support nursing competence was 71 (62, 74) and nutrition nursing related quality scores 60 (53.75, 62).

**Table 2:** The scores and standard score rate of each dimension of nutritional care competence scale for clinical nurse (*n* = 1,218).

Variables	No. of item	Score ranges	Score	Item mean score
Nutrition expertise	7	10 ~ 35	25 (22, 28)	4 (3, 4)
Clinical nutrition assessment ability	10	13 ~ 49	37 (27, 40)	4 (3, 4)
Nutrition related health education capacity	13	23 ~ 62	48 (40, 52)	4 (3, 4)
Nutrition support nursing capacity	18	36 ~ 87	71 (62, 74)	4 (3, 4)
Nutrition and nursing related qualities	15	28 ~ 68	60 (53.75, 62)	4 (3, 4)
Nutritional nursing ability	63	126 ~ 300	238 (210, 253)	

### 3.3 Single factor analysis of relate to nutritional nursing competence of clinical nurses

The results of this study showed that educational background, professional title, hospital grade, department, monthly income, region, learning nutrition courses in school, participating in continuing education and training, nutrition specialist nurses, participating in nutrition and nursing related competitions, regular nutrition quality inspection in hospitals, and the implementation of nutrition and nursing quality improvement projects in hospitals had an impact on the nutritional nursing competence of clinical nurses(*p*<0.05), as shown in Table 1.

### 3.4 Multiple linear regression analysis of nutritional nursing competence of clinical nurses

The total score of nutrition nursing competence of clinical nurses was taken as dependent variable, and the variables with statistical significance in univariate analysis were taken as independent variables. The assignment of independent variables was shown in Table 3. The input method was used to filter variables and perform multiple linear regression analysis. The results are shown in Table 4. The tolerance of each model is less than 1, and the variance inflation factor is less than 5, indicating that there is no multicollinearity between the independent variables. Finally, the factors that predict the nutritional nursing competence of clinical nurses are as follows: (1) Hospital level, (2) department, (3) region, (4) studied nutrition courses in school, (5) participated in continuing nutrition education and training, (6) was a nutrition nurse, (7) participated in nutrition nursing related competitions, (8) The hospital regularly carried out nutrition related nursing special inspection, (9) The hospital carried out nutrition related nursing quality improvement projects.

**Table 3:** Self-variable assignment.

Independent variables	Reassignment
Education background	College or below = (0,0,0) Undergraduate = (0,1,0) Master or above = (0,0,1)
Professional title	Master or above = (0,0,0,0,0) Nurse = (0,1,0,0,0) Junior nurse = (0,0,1,0,0) Registered nurse = (0,0,0,1,0) Senior nurse = (0,0,0,0,1)
Hospital level	Second class B and below = (0,0,0,0) Second class A = (0,1,0,0) Third class B = (0,0,1,0) Third class A = (0,0,0,1)
Department	Internal medicine = (0,0,0,0,0,0) Surgical department = (0,1,0,0,0,0) Gynecology and obstetrics = (0,0,1,0,0,0) Pediatrics department = (0,0,0,1,0,0) ICU = (0,0,0,0,1,0) Other = (0,0,0,0,0,1)
Monthly income	≤3,000 = (0,0,0,0) 3,001 ~ 5,000 = (0,1,0,0) 5,001 ~ 8,000 = (0,0,1,0) >8,000 = (0,0,0,1)
Region	East region = (0,0,0) Central region = (0,1,0) West region = (0,0,1)
Learn nutrition courses in school	Yes = (1) No = (0)
Participate in nutrition related continuing Education and training	Yes = (1) No = (0)
Be a nutrition specialist nurse	Yes = (1) No = (0)
Participate in nutrition nursing related Knowledge/skills competition	Yes = (1) No = (0)
Hospital regularly carries out special Examination of nutrition nursing	Yes = (1) No = (0)
Carry out nutrition and nursing special quality Improvement projects	Yes = (1) No = (0)

**Table 4:** Variables related nurses’ nutritional care competence (multivariated stepwise regression analysis, *N* = 1,218).

Variables	*B*	*SE*	*β*	*t*	*p*	95%*CI*
Constant	324.828	12.197		26.632	0	300.898 ~ 348.758
Undergraduate	−0.288	2.867	−0.003	−0.101	0.92	−5.913 ~ 5.336
Master or above	9.22	11.651	0.021	0.791	0.429	−13.64 ~ 32.079
Junior nurse	4.754	3.396	0.062	1.4	0.162	−1.909 ~ 11.418
Registered nurse	3.788	3.695	0.049	1.025	0.306	−3.462 ~ 11.038
Senior nurse	4.917	5.318	0.031	0.925	0.355	−5.517 ~ 15.351
Second class A	6.608	4.792	0.061	1.379	0.168	−2.795 ~ 16.011
Third class B	12.475	4.67	0.128	2.671	0.008	3.313 ~ 21.638
Third class A	11.728	5.015	0.152	2.338	0.02	1.888 ~ 21.568
Surgical Department	0.125	2.607	0.001	0.048	0.962	−4.989 ~ 5.239
Gynecology and Obstetrics	−7.721	4.521	−0.046	−1.708	0.088	−16.59 ~ 1.148
Pediatrics Department	−2.474	4.525	−0.015	−0.547	0.585	−11.351 ~ 6.403
ICU	−9.891	3.001	−0.095	−3.296	0.001	−15.779 ~ −4.004
Other	−7.341	3.001	−0.075	−2.446	0.015	−13.23 ~ −1.453
Central region	−0.523	3.07	−0.007	−0.17	0.865	−6.546 ~ 5.499
West region	−6.204	2.849	−0.081	−2.178	0.03	−11.793 ~ −0.615
3,001 ~ 5,000	2.517	3.456	0.033	0.728	0.467	−4.263 ~ 9.297
5,001 ~ 8,000	6.883	3.746	0.088	1.837	0.066	−0.467 ~ 14.234
>8,000	8.476	4.549	0.074	1.863	0.063	−0.449 ~ 17.402
Learn nutrition courses in school	−8.951	1.95	−0.119	−4.59	0	−12.778 ~ −5.125
Participate in nutrition related continuing education and training	−13.211	2.184	−0.172	−6.049	0	−17.496 ~ −8.926
Be a nutrition specialist nurse	−12.13	5.189	−0.062	−2.338	0.02	−22.311 ~ −1.949
participate in nutrition nursing related knowledge/skills competition	−8.614	3.204	−0.072	−2.688	0.007	−14.901 ~ −2.327
Hospital Regularly carries out special examination of nutrition nursing	−9.38	2.69	−0.124	−3.487	0.001	−14.657 ~ −4.102
Carry out nutrition and nursing special quality improvement projects	−10.411	2.625	−0.138	−3.966	0	−15.561 ~ −5.261

*R = 0.517, R2 = 0.268, F = 18.178 p<0.001.*

## 4 Discussion

The purpose of this study was to evaluate the status quo of nutritional nursing competence of Chinese nurses and explore its influencing factors. The nutritional nursing competence is in the middle level, which is consistent with the results of Zhu Xinyi (31) and Li Haiqun (32), and needs to be further improved. Clinical nurses play an important role in protecting patients’ health and improving their quality of life (29, 33). Therefore, providing nutritional care to patients requires investigating the level of nutritional care competence of nurses to ensure that accurate and targeted nutritional care knowledge is provided to patients. At present, most studies mainly focus on the professional knowledge and literacy of nutrition, and less attention is paid to the nutrition education and implementation of nutrition nursing procedures for patients by nurses, resulting in the knowledge can not be effectively translated into clinical practice (33, 34). Therefore, in this case, nurses’ clinical nutrition nursing competence involves mastering nutrition professional knowledge, cultivating nutrition literacy, comprehensive nutrition assessment, nutrition health education and nutrition nursing practice, so as to meet the basic nutritional nursing needs of patients in various disease states and social and cultural backgrounds.

The hospital grade and work department of clinical nurses are the factors affecting nutrition nursing. This study only discusses the nutritional nursing competence of nurses in different departments in the clinical front line, and does not involve the administrative and logistical nursing staff. In this study, the higher the level of hospital, the higher the level of nutritional nursing competence of nurses. Nurses in tertiary hospitals have more opportunities to obtain professional nutrition training and become nutrition specialist nurses (35). In addition, due to the higher disease severity of patients in tertiary hospitals, nurses are required to have more perfect nutritional nursing competence to meet the diversified nutritional needs of patients (36). Most ICU patients have fasting, chewing difficulties and malnutrition (37). Nurses play a leading role in the implementation of nutrition nursing, and the competence of nutrition nursing is higher than that of other departments such as internal medicine. Intensive nutritional support therapy for patients admitted to ICU can supplement the energy required by the body and enhance physical fitness, which is one of the important treatment methods to promote the recovery of patients (38).

In this study, nurses with specialized nutrition or those with systematic and standardized nutrition nursing education and training in school or work had better nutrition nursing level. Professional nutrition courses and standardized nutrition nursing continuing education can not only learn nutrition-related knowledge, but also understand the cutting-edge nutrition nursing concepts and new technologies, and constantly improve the level of nutrition knowledge, attitude and behavior of nurses (36). At present, the school education only mentions the related nutrition nursing knowledge in some chapters of the nursing course, and there is no special nutrition nursing courses. In the continuing education of in-service nurses, for example, the nutrition specialist nurses of the Chinese Nursing Association and the nutrition specialist nurses of various provinces will systematically study relevant courses. Therefore, schools can improve students’ interest and enthusiasm in learning nutrition courses by setting up scientific and reasonable nutrition courses and diversifying teaching modes. In addition, multiple nutrition nursing learning channels are provided to clinical nurses to meet the nutritional knowledge needs of nurses and patients (39). Li (40) confirmed that the qualified rate of nurses’ nutrition knowledge score increased from 51.02 to 73.47% through standardized nutrition training. It is indispensable that the nutrition training of clinical nurses should be in line with the needs and diseases of patients, so that nutritional nursing practice can be truly implemented in patients and benefit patients.

This study showed that regular nutritional nursing quality inspection and nutritional nursing quality improvement programs in hospitals are beneficial to improve the nutritional nursing competence of clinical nurses. The hospital set up a special quality improvement group for nutrition nursing, which can supervise and analyze the problems existing in clinical nutrition nursing, and improve the nutrition knowledge and skills of nurses (32). Carry out nutrition and nursing quality improvement projects, strengthen knowledge and skills training related to nutrition and nursing, reduce the construction of wrong nutrition and nursing practices due to insufficient nutrition knowledge, and ensure the safety of nutrition and nursing.

Through this questionnaire survey, the total score of clinical nurses’ nutrition nursing competence and the scores of each dimension were obtained, which directly reflected the ability level of nurses in nutrition nursing. In addition, the study also compared the scores of nutrition nursing competence of nurses with different characteristics (such as years of work, education, professional titles, departments, etc.), revealing the differences of nutrition nursing competence among different groups. These differences help to identify certain existing factors that may have a positive or negative impact on nurses’ ability to care for nutrition. Compared with other studies, the advantages of this study are as follows: First, the sample size of this study is large, and it covers different regions and different types of hospitals, which improves the representativeness and universality of the research results. Secondly, through multiple linear regression analysis, the main factors affecting nurses’ nutrition nursing competence can be accurately identified, which provides a scientific basis for the formulation of targeted improvement measures. Most importantly, the study adopted a multi-dimensional nutrition nursing competence scale, which can comprehensively assess nurses’ nutrition nursing competence, avoiding the limitation of a single dimension.

There are some limitations in this study. Firstly, the study was only conducted in cities in mainland China, so the promotion to other countries and regions may be limited. The research on the status quo and influencing factors of nutrition nursing ability of Chinese nurses has certain adaptability to other international environments, but it needs to be analyzed in detail on the basis of considering cultural differences, differences in medical systems and the universality of influencing factors. In order to improve the nutritional nursing capacity of nurses around the world, countries should strengthen international cooperation and exchanges to jointly promote the training and education of nutritional nursing. At the same time, countries should also formulate targeted policies and measures according to their own conditions to improve the professional competence and service quality of nurses. Secondly, the scale used in this study was self-reported by participants, without objective measurement, so the results may be biased. Finally, Cross-sectional studies can only reveal the state of the respondents at a certain point in time, and cannot determine the causal relationship between factors and results. Therefore, the results of the study may be affected by time factors and cannot fully reflect the dynamic change of nurses’ nutritional nursing competence. The study did not carry out long-term follow-up observation, which could not understand the long-term trend of nurses’ nutritional nursing competence and the effect of improvement measures.

## 5 Conclusion

The nutrition nursing of clinical nurses in China is at a medium level. The nutrition nursing competence of registered nurses in China is affected by department, hospital level, being a nutrition specialist nurse, in-school and continuing education and training, regular supervision and implementation of nutrition nursing quality improvement projects in hospitals. Our findings suggest that schools and hospitals should pay more attention to the cultivation of nutritional nursing, and strengthen and train nurses to improve the level of nutritional nursing.

## Data Availability

The original contributions presented in the study are included in the article/supplementary material, further inquiries can be directed to the corresponding authors.
